# Advanced neuroprosthetic electrode design optimized by electromagnetic finite element simulation: innovations and applications

**DOI:** 10.3389/fbioe.2024.1476447

**Published:** 2024-11-06

**Authors:** Shu Yang, Siyi Yang, Peixuan Li, Shuchun Gou, Yuhang Cheng, Qinggang Jia, Zhanhong Du

**Affiliations:** ^1^ Guangdong Provincial Key Laboratory of Brain Connectome and Behavior, CAS Key Laboratory of Brain Connectome and Manipulation, The Brain Cognition and Brain Disease Institute (BCBDI), Shenzhen-Hong Kong Institute of Brain Science, Shenzhen Institute of Advanced Technology, Chinese Academy of Sciences, Shenzhen, China; ^2^ Faculty of Life and Health Sciences, Shenzhen University of Advanced Technology, Shenzhen, China; ^3^ Shenzhen Fundamental Research Institutions, Shenzhen, China; ^4^ University of Chinese Academy of Sciences, Beijing, China; ^5^ Institute of Applied Physics and Computational Mathematics, Beijing, China

**Keywords:** neuroprosthesis, neural electrode, finite element model, neuron simulation, neuroprosthesis simulation

## Abstract

Based on electrophysiological activity, neuroprostheses can effectively monitor and control neural activity. Currently, electrophysiological neuroprostheses are widely utilized in treating neurological disorders, particularly in restoring motor, visual, auditory, and somatosensory functions after nervous system injuries. They also help alleviate inflammation, regulate blood pressure, provide analgesia, and treat conditions such as epilepsy and Alzheimer’s disease, offering significant research, economic, and social value. Enhancing the targeting capabilities of neuroprostheses remains a key objective for researchers. Modeling and simulation techniques facilitate the theoretical analysis of interactions between neuroprostheses and the nervous system, allowing for quantitative assessments of targeting efficiency. Throughout the development of neuroprostheses, these modeling and simulation methods can save time, materials, and labor costs, thereby accelerating the rapid development of highly targeted neuroprostheses. This article introduces the fundamental principles of neuroprosthesis simulation technology and reviews how various simulation techniques assist in the design and performance enhancement of neuroprostheses. Finally, it discusses the limitations of modeling and simulation and outlines future directions for utilizing these approaches to guide neuroprosthesis design.

## 1 Introduction

### 1.1 Enhancing neuroprosthesis development through simulation techniques

The human nervous system supports cognitive functions such as cognition, decision-making, and consciousness (Yuste, 2015; Qi et al., 2019; Zhu et al., 2020). Neurological damage often permanently impairs physiological functions—such as causing paralysis following spinal cord injury or impairing speech and motor functions after a stroke—with these disabilities persisting throughout the patient’s lifetime, self-repair is essentially impossible. In theory, interventions such as rehabilitation (Al’joboori et al., 2020) and nutrient injections, polymer scaffold or stem cells into affected areas (Cofano et al., 2019; Al’joboori et al., 2020; Huang et al., 2020) may induce some degree of neural regeneration; however, these approaches remain largely experimental and have not demonstrated robust effectiveness in clinical applications.

Electrophysiological-based neuroprostheses offer a direct means to reconstruct damaged nervous systems and facilitate the restoration of neural functions through human-machine symbiosis (Krucoff et al., 2016; Du et al., 2017b; Zhanhong Du et al., 2020; Li et al., 2023). Typically employing neural interfaces (Vakilipour and Fekrvand, 2024), these devices encode and decode neural electrical signals, enabling interaction between external electronic systems and the nervous system (Kozai et al., 2014; Du et al., 2015; Kolarcik et al., 2015). The effectiveness of neuroprostheses hinges not on the regeneration and repair of neural tissue but on their design, which should prioritize minimal invasiveness, high efficiency, and biological safety. These criteria define the future trajectory for the development of neuroprostheses.

**FIGURE 1:**
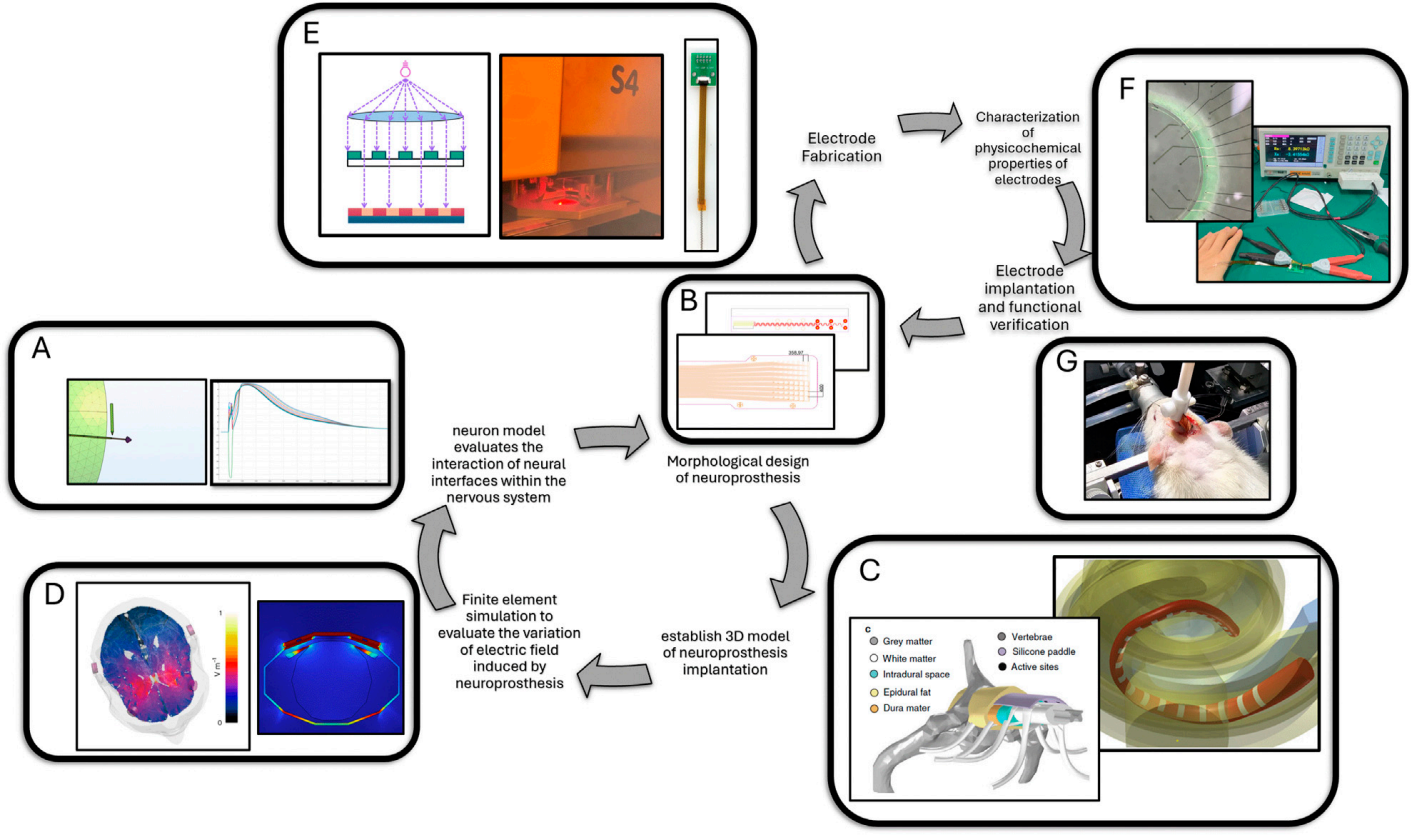
Schematic diagram illustrating the development of neural electrodes driven by modeling and simulation. Image in **(C)** (Nogueira et al., 2016; Greiner et al., 2021). Image in (Vakilipour and Fekrvand, 2024) **(D)** (Vassiliadis et al., 2024). **(A)** Neuronal modeling and simulation. **(B)** Electrode two-dimensional structure modeling. **(C)** Three-dimensional modeling of electrodes and tissues. **(D)** Finite element simulation of electromagnetism in electrodes and tissues. **(E)** Micro and nano-manufacturing of neural electrodes. **(F)** *In vitro* testing of electrode performance. **(G)** Animal implantation testing of electrode performance.

Modeling and simulation can guide the design of neuroprostheses in two main ways. Figure 1 illustrates the specific processes by which modeling and simulation technologies accelerate the development of neural electrodes. Firstly, through numerical calculations, modeling and simulation can simulate the interaction between neuroprostheses of various shapes and implantation sites with the nervous system (Chen et al., 2017; Song E. M. et al., 2020; Leuthardt et al., 2021; Fekete et al., 2023). During the simulation process, the design can be optimized to minimize invasiveness while maximizing the information collected and input by the neuroprosthesis (Du et al., 2019). Additionally, using modeling and simulation technologies provides a virtual environment for electrode implantation testing, which reduces the number of iterations from design to *in vivo* validation and shortens the development cycle of neuroprostheses.

## 2 Neuroprosthesis simulation model

### 2.1 Building simulation models from real physiological structures

**FIGURE 2:**
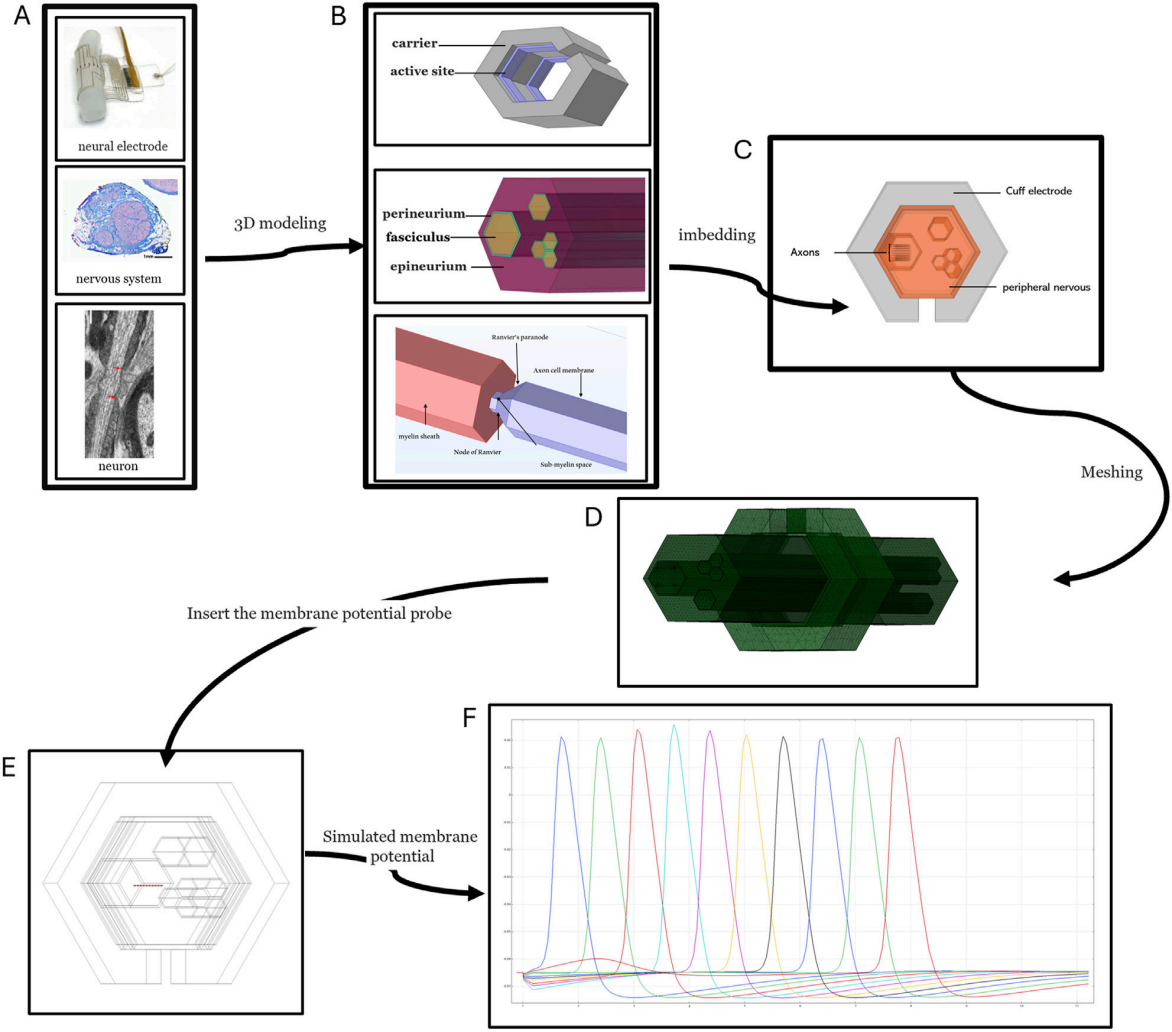
Process for establishing and simulating a model of interaction between neural electrodes and the nervous system. The image in **(A)** (Settell et al., 2020; Dustin et al., 2023; Paggi et al., 2024). **(A)** Neural electrodes, nerves, neuronal structures. **(B)** Discrete three-dimensional models of neural electrodes, nerves, and neurons. **(C)** Combined three-dimensional models of neural electrodes, nerves, and neurons. **(D)** Finite element mesh division of combined three-dimensional models of neural electrodes, nerves, and neurons. **(E)** Insertion of probes into the simulation model of neural electrodes, nerves, and neurons. **(F)** Schematic diagram of the simulation results of membrane potential (action potential) at the probe location.

The neuroprosthetic interaction model with neurons in biological tissues and systems comprises three primary components: the neuroprosthesis itself, the physiological environment, and the neurons. Each of these components requires individual modeling before integration into a cohesive framework. Figure 2 provides a brief process flow from the physical object to the modeling and simulation of neural electrodes and tissues. The modeling process is structured into five hierarchical levels:

Organ Level: This foundational level involves defining the geometry of the electrode implantation site, its relationship with the target organ, and the morphology of surrounding physiological tissues, including the brain’s surface, dura mater, cerebrospinal fluid, bones, muscles, and fat. Tools such as CT scans (Thompson et al., 2020; Anjum et al., 2022), MRI (Cohen-Adad et al., 2021a; Cohen-Adad et al., 2021b), and anatomical atlases (Puljak et al., 2009) are instrumental in acquiring detailed organ shape data. Notably, CT and MRI offer non-invasive insights into internal organ structures, which are invaluable for personalized applications, such as tailor-made cochlear implant electrodes or customized spinal epidural electrodes (Nogueira et al., 2016; Greiner et al., 2021).

The second level is the tissue level, where the conductivity and relative permittivity of the electrodes and each tissue need to be determined. Some tissues, such as white matter, have large anisotropy in their conductivity and relative permittivity, which should ideally be reflected in the model’s physical parameter settings during the modeling process.

The third level is the cell group level. Taking peripheral nerve modeling as an example, the data that need to be determined are the types of nerve fibers within each nerve bundle, the percentage of each type of fiber and its distribution location, the orientation of the different nerve fibers, the types of signals transmitted, and the peripheral nerves and target organs (e.g., spinal epidural stimulation model) among the need to quantify the control of muscle contraction by spinal epidural electrical stimulation through the percentage of activated nerve types, and the target organs that are controlled by the nerves (Greiner et al., 2021). The distribution of neuron types under different cortical thicknesses needs to be clarified for cortical modeling.

Electromagnetic field finite element models are commonly used for modeling organs, tissues, and implanted neuroprostheses. Given that the frequency of electrical stimulation and neuronal firing rates generally do not exceed 50 kHz, electromagnetic induction effects are typically negligible. Therefore, models commonly utilize an electro-quasistatic field, which excludes magnetic effects (Steinmetz et al., 2006; Bossetti et al., 2008).

The fourth level is the neuron level, where the structure of neurons needs to be specified. The modeling of peripheral nerves only needs to consider the structure of the axon, such as the positional distribution of Longfellow’s node, its length, diameter, and the passive impedance properties of the cell membrane. Cellular modeling of the central nervous system is much more complex because the neural skeleton and shape are more sophisticated. In the CNS, it is necessary to reconstruct the skeleton (Abdellah et al., 2018; Wang X. J. et al., 2021; Xu et al., 2021) of the nerve cells based on microscopic scans and the neural structures in the tissues across scales. MRG modeling of neurons requires a neural skeleton structure. For finite element modeling of neurons, the neuronal shape data that can be used for finite element modeling usually needs to be reconstructed into a 3D model of the neuronal skeleton before it can be obtained. Open-source frameworks such as NeuroMorphoVis (Abdellah et al., 2018) from BlueBrain provide the operational paradigm. These frameworks can reconstruct neural 3D watertight models based on neuronal skeleton structure data. However, such reconstructions are not strictly realistic, which may pose a challenge to the predictive realism of the model. 2024 In a recent study, scientists reconstructed a cubic millimeter of the temporal cortex. The model contains about 57,000 cells and 150 million synapses (Shapson-Coe et al., 2024), and these spectral maps, connectomics, and cell morphology studies may all provide valuable information for future neurophysiological simulations.

The fifth level is the ion channel level, where the types of ion channels on the axon and the kinetic equations for each channel must be clarified. In addition, the density of the distribution of ion channels on each subcellular unit of the neuron and the differences in the distribution of ion channels at different locations (e.g., Rumphius node, axon initial segment, dendrites, and nerve endings), resulting in different membrane dynamics in different subcellular units, also need to be clarified (Lai and Jan 2006; Mercer et al., 2007; Schmidt-Hieber and Bischofberger, 2010; Hallermann et al., 2012). Most differential kinetic equations of ion channels required for modeling are open-source at ModelDB (Hines et al., 2004).

**FIGURE 3:**
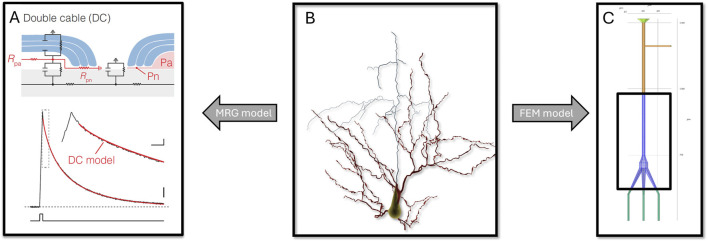
From the neuron morphological model **(B)** to the neuron MRG model [**(A)** (Cohen et al., 2020)] and the finite element mesh model **(C)**.

Neuronal models typically fall into two categories: those based on the Hodgkin-Huxley (HH) model, known as MRG (McIntyre-Richard-Grill) models, and those constructed using the finite element method. Both types involve simplifications of the actual neuronal structures. Figure 3 shows the simplification process of the three-dimensional structure of a neuron into the MRG model and the neuron finite element model in terms of structure.

Electrophysiological modeling of neuron is a critical step in developing a simulation model for the interaction with the nervous system after a neuroprosthesis has been implanted. The MRG model views neurons as one-dimensional cables, with their internal and external environments modeled as series-connected resistors (McIntyre et al., 2002). In contrast, the finite element model treats these environments as physical fields, typically modeled as quasi-static electric fields (Fellner et al., 2022) and based on ion diffusion dynamics (Mori et al., 2008). The phospholipid bilayer, the main component of the membrane, acts as a dielectric material with a specific capacitance, which is consistent across different types of neurons and relatively easy to measure. Membrane resistance, however, varies widely depending on the neuron type and location, and can change dynamically with voltage or external conditions such as extracellular electric fields (Lai and Jan 2006; Schmidt-Hieber and Bischofberger, 2010), light (Diester et al., 2011; Gong et al., 2020), mechanical forces (Brohawn et al., 2019; Douguet and Honoré, 2019), and chemical stimulation (Du et al., 2017a; Flavin et al., 2022).

Myelin, produced by oligodendrocytes, wraps around neuron axons and plays a crucial role in enhancing the speed and fidelity of action potential conduction. In the MRG model, myelin is represented as a series resistor with low conductivity. The finite element model, however, treats it as a material with high resistivity, modeling action potentials as propagating in jumps through myelinated segments, with significant weakening in regions without ion channels. In a 2019 study, it was confirmed that the longitudinal conduction pathway in the sub-myelin region assumes an essential role in reproducing the spatiotemporal distribution of action potentials by modeling in comparison with membrane clamps and optical recordings (Cohen et al., 2020).

### 2.2 Mainstream simulation frameworks and their application scenarios

Currently, a diverse array of simulation frameworks is utilized to guide the design of neuroprostheses by simulating interactions with neurons within biological tissues. These frameworks generally fall into two categories: hybrid models (Romeni et al., 2020) and comprehensive finite element models (Lopreore et al., 2008; Mori et al., 2008; Fellner et al., 2022). The primary difference between these lies in the neuronal modeling approach—either using the MRG model or a finite element model (McIntyre et al., 2002). Table 1 summarizes the advantages and disadvantages of the neuron's MRG model and FEM model.

**TABLE 1:** Comparison of the advantages and disadvantages between the MRG neuron model and the finite element neuron model.

	Neuron MRG Model	Neuron Finite Element Model
Applicability	Suitable for one-dimensional axons and simple structures	Suitable for any complex structure
Accuracy	High accuracy for one-dimensional problems	High accuracy, affected by mesh quality
Computational Efficiency	Low computational cost, high efficiency	High computational cost, low efficiency
Implementation Difficulty	Simple model, easy to implement	Complex model, requires professional software support
Spatial Heterogeneity	Difficult to handle	Can be easily handled
Extensibility	Difficult to extend to complex geometries	Easy to extend, highly adaptable

Hybrid models combine finite element methods to simulate electrical conduction within physiological tissues under stimulation. They compute voltage at each spatiotemporal point, which is subsequently fed into a neuron cable model. This model predicts the potential generation of action potentials by neurons. It’s important to note that this simulation process is unidirectional, meaning it does not consider the feedback effects of neuron-generated action potentials on the surrounding tissues. Additionally, the neuron cable model in hybrid frameworks is essentially one-dimensional, which could restrict its ability to predict neurons' real-time behavior under electrical stimulation accurately. The neuron’s finite element model can establish more realistic models of myelinated or unmyelinated neurons. These models are established in the simulation process along with the extracellular tissue space and the neuroprosthesis using the same physical field, so they can inherently be coupled together, simulating the real-time interaction between the neuron and its surrounding microenvironment. Additionally, they can provide a detailed description of the occurrence, conduction, and extinction of action potentials on the three-dimensional surface occupied by the neuron’s cell membrane, displaying many characteristics not possessed by the neuron’s MRG model, such as describing the transmembrane diffusion of various ions during an action potential and some subtle waveform changes during the action potential generation process.

Despite these constraints, hybrid models are widely used for predicting neural behavior under electrical stimulation and the changes in the electrical field triggered by neural activity. They are applied in various settings, including peripheral nerve stimulation (Raspopovic et al., 2017), epidural spinal stimulation (Greiner et al., 2021), cortical microstimulation (Kumaravelu et al., 2022), transcranial electrical stimulation (Grossman et al., 2017), and deep brain stimulation (Åström et al., 2015). These applications are crucial for both exploring underlying mechanisms and providing experimental guidance. However, because the neuron’s finite element model simulation consumes more computational resources compared to the neuron’s MRG model simulation, this limits the application scope of the full finite element model. Currently, most studies on full finite element models are based on single neuron finite element models for small-scale expansion. No studies attempt to embed many neurons simultaneously in the full finite element model, as expanding the model’s scale and increasing the number of neurons both increase computational complexity, which was difficult to meet with computational requirements years ago. In the future, as computer computing power continues to improve, making it possible to support the simultaneous simulation of hundreds or even thousands of neurons in full finite element models.

In practice, several well-established modeling and simulation systems support neuroprosthetic development. For instance, Sim4Life (Fasse et al., 2024; Vassiliadis et al., 2024) is tailored for spinal cord and brain modeling, PyPNS(Lubba et al., 2019) is suited for peripheral nerve modeling, and ASCENT (Musselman et al., 2021) is used for additional applications. These systems, along with combinations of Comsol and NEURON software, are integral in advancing neuroprosthetic design and simulation (Hines and Carnevale, 1997; Romeni et al., 2020).

## 3 Neural interface simulation applications

According to their invasiveness, brain-computer interfaces (BCIs) can be categorized into three types: invasive, partially invasive, and non-invasive (Vakilipour and Fekrvand, 2024). Invasive BCIs are directly embedded in the cortex during neurosurgical procedures, allowing for the monitoring of individual neuronal activity. Partially invasive brain interfaces use electrocorticography, which involves electroencephalographic (EEG) recordings made with intracranial subdural or depth electrodes. Smaller surgical openings in the brain significantly reduce their invasiveness (Vakani and Nair, 2019) Non-invasive BCIs utilize external detectors rather than brain implants, thus eliminating the need for surgical intervention. To mitigate the long-term damage associated with invasive neural interfaces, flexible BCIs are becoming a focal point of research (Du et al., 2017b). Common flexible conductive layers include 1) ultra-thin noble metal wires, such as gold and platinum, 2) liquid metals like gallium and bismuth, and 3) conductive polymers such as poly (3,4-ethylenedioxythiophene) polystyrene sulfonate (PEDOT), polypyrrole (PPy), polyaniline (PANI), polythiophene (PT), and their derivatives (Remy et al., 2024; Ji et al., 2023). These common conductive layers are limited by the electrical and mechanical properties of their base materials and do not adequately meet existing demands, leading to the further composite construction of new flexible conductive layers (Wang et al., 2017). Moreover, next-generation conductive nanomaterials such as graphene and MXene are also being explored for the development of flexible conductive layers with unique micro-nano structures (Gou et al., 2024).

### 3.1 Brain-machine interface

#### 3.1.1 Quantitative analysis of extracellular action potentials

**FIGURE 4:**
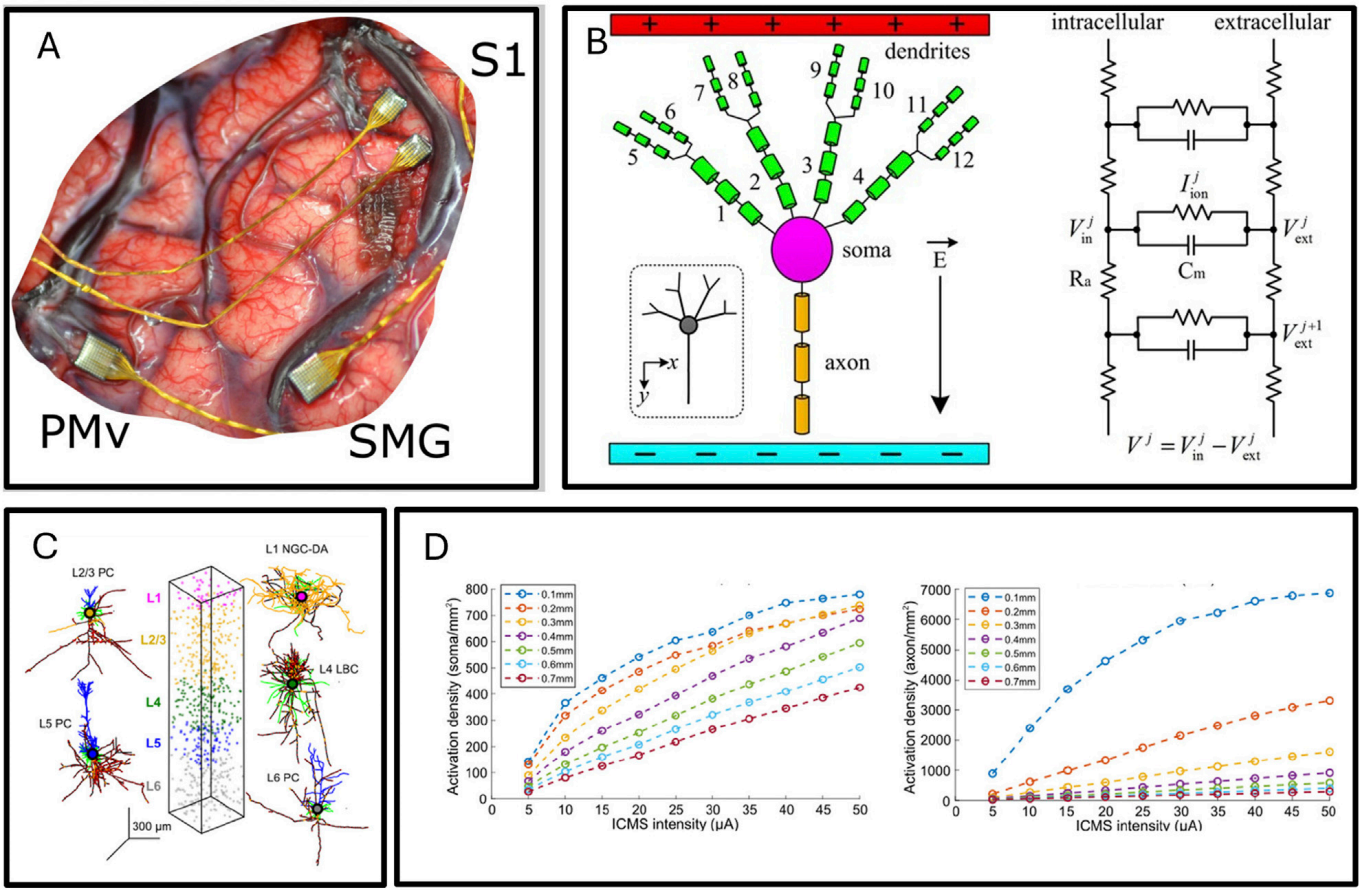
**(A)** high-density electrode array implanting in the cerebral cortex (Salas et al., 2018). **(B)** Artificial pyramidal neuron, from multi-compartment model to circuit model (Yi et al., 2017). **(C)** Mathematical model of cerebral cortex cells, including neuron types, spatial arrangement characteristics. **(D)** The proportion of action potential generated by activation of axons and cell bodies of different diameter under different cortical microstimulation amplitude (Kumaravelu et al., 2022)**.**

Invasive brain-machine interfaces, by implanting electrodes within the cortex, can directly record single neural action potentials (pulses), providing the largest amount of decoded information among brain-machine interfaces. Figure 4A shows the implantation of a cortical electrode. Most invasive brain-machine interfaces are based on high-density rigid silicon electrodes like blackarray and neuropixel, and have been widely used in neuroscience research such as decoding motor intentions, cortical microstimulation, and language decoding. Flexible, high-density invasive electrodes represent the next-generation of invasive neural interfaces. Compared to rigid silicon electrode arrays, flexible electrodes cause less damage to neural tissue due to displacement after implantation, and many brain grooves and gyri, which were previously difficult to implant with rigid neural interfaces, can now be implanted with flexible electrodes. Currently, neurolink has implanted multi-site flexible electrodes in multiple volunteers, and some volunteers have been able to play very popular games like Civilization VI and Mario Kart 8 (https://www.nme.com/news/gaming-news/watch-elon-musks-first-neuralink-patient-play-mario-kart-with-his-mind-3610693) through the brain-machine interface, although there have also been reports of electrode dislodgement risks.

The reason why invasive brain-machine interfaces extract a greater amount of brain activity information than cortical electrodes (ecog) and EEG is that invasive brain-machine interfaces can record the extracellular electric field changes induced by the firing of neural action potentials. Currently, there are no robust methods to achieve intracellular recordings of neurons *in vivo*. Even high-resolution neural electrode arrays with front-end amplifiers like neuropixel can only record extracellular action potentials, but recording single neurons' *in vivo* activity can be used to analyze neural microcircuits (Happel et al., 2010). If accurate recording of signals to infer the activity state of a single neuron through the neural interface is desired, tracing the source of extracellular action potentials is necessary (Somogyvári et al., 2012; Cserpán et al., 2017). One method is to first use the cable model to calculate the spatiotemporal transmission process of the action potential along the neuron’s morphology, then use the diffusion model of the current to calculate the waveform of the extracellular action potential at different positions (Gold et al., 2006). Experiments have proven that the model simulation data basically match the experimental recording data, and this model simulation research has greatly advanced the analysis of action potential tracing.

#### 3.1.2 Accurate prediction of action potential activation by cortical microstimulation

Cortical microstimulation generally uses rigid, high-density silicon electrode arrays implanted into the cortex to stimulate and activate neurons. Cortical microstimulation is commonly used to restore sensation by precisely applying electrical stimulation in time and space by implanting cortical microstimulation electrodes into the sensory cortex, simulating real sensory-induced cortical activity. Cortical microstimulation electrodes combined with automatic control systems, somatosensory encoding and decoding systems, and mechanical arms connected to tactile sensors can achieve bionic tactile mechanical arm control. A research team represented by the University of Pittsburgh demonstrated in a study published in 2013 on macaques through cognitive neuroscience experiments that tactile restoration using a cortical microstimulation brain-machine interface with a prosthetic hand is feasible (Tabot et al., 2013). A study published in 2016 confirmed that this strategy is also feasible in humans (Flesher et al., 2016). A study published in 2021 reported the use of a sensory-motor loop brain-machine interface to achieve real-time sensory feedback brain-controlled mechanical arm control (Flesher et al., 2021). Besides somatosensory sensation, cortical microstimulation electrodes are also widely used in reconstructing vision (Chen et al., 2020). Although cortical microstimulation has many applications, many details about its functioning remain unclear. For example, the spatial effects of ICMS are still controversial: Stoney and colleagues proposed that the amount of somatic activation increases with stimulation intensity, while Histed and others believe that the density of activation (rather than the amount of somatic activation) increases with stimulation intensity. In a study published in 2021, researchers based on the cortical column computational model analyzed the activation of cortical neurons around the electrode with increasing stimulation amplitude. In Figures 4B, C, the modeling of neurons and the arrangement of neurons within cortical units in this modeling process are shown, and the computation result shows in Figure 4D. The simulation results showed that under all amplitudes, the main mode of somatic activation is axonal activation followed by retrograde propagation to the soma, rather than through synaptic activation. Direct activation of the soma or dendrites did not occur. The volume of cortex producing retrograde action potentials increased with stimulation amplitude, while the volume of soma activation increased slightly. However, the density of soma activation within the activated volume increased with stimulation amplitude. The volume of cortex producing action potentials increased with increasing ICMS amplitude, consistent with Stoney’s view. However, the volume occupied by activated somas remained roughly constant, while the density of neurons activated within that volume increased, consistent with Histed’s view (Kumaravelu et al., 2022).

### 3.2 Epidural spinal nerve interface

The research team led by Grégoire Courtine at the Swiss Federal Institute of Technology in Lausanne has achieved breakthroughs in the field of motor recovery using epidural paddle electrodes from rodents to primates to humans over the past decade. By implanting paddle electrodes in the epidural space of the spinal cord and applying electrical stimulation at certain frequencies and intensities to the spinal dorsal roots, the central pattern generators in the spinal cord can be activated to restore the patient’s motor function. In 2018, spinal cord injury patients needed several months of practice combined with assistive standing systems to complete walking activities after implanting spinal epidural electrodes (Wagner et al., 2018). In 2022, researchers increased the active sites of epidural electrodes, updated the encoding method, and used modeling to customize the design of the electrodes, greatly improving the targeting of epidural electrical stimulation. This allowed the electrodes to activate specific sensory nerves in a single dorsal root more concentratedly, ultimately enabling implanted electrode patients to complete standing, stepping, and pedaling special bicycles and other rhythmic walking activities within a few days. In 2016, the research team established a combined recovery system of motor cortex brain-machine interface and spinal epidural electrical stimulation on a spinal transected macaque (Capogrosso et al., 2016). The motor commands extracted from the motor cortex controlled the discharge of the spinal epidural electrodes, ultimately allowing the macaque to control the start, stop, and stepping speed through the cortical electrodes. In a study published in 2023, a similar function was achieved in spinal cord injury patients using a brain-spinal cord neural interface combining less invasive ecog (Lorach et al., 2023). In the same year, the research team used a deep brain electrode-spinal cord electrode closed-loop system to successfully improve the gait disorder of a refractory Parkinson’s patient (Milekovic et al., 2023).

The spinal epidural electrode stimulation system can also be applied to upper limb motor function recovery. In 2022, the research team studied spinal epidural electrical stimulation recovery of upper limb motor function. They implanted customized electrodes in the partially transected (20%–40%) cervical spinal cord of macaques at the C5-C6 segments. By guiding the control of the active site targets of the electrodes through simulation, they demonstrated that specific forms of electrical stimulation could significantly enhance the efficiency of spinal cord injury macaques in grasping objects, proving the feasibility of epidural stimulation in restoring upper limb motor ability (Barra et al., 2022).

**FIGURE 5:**
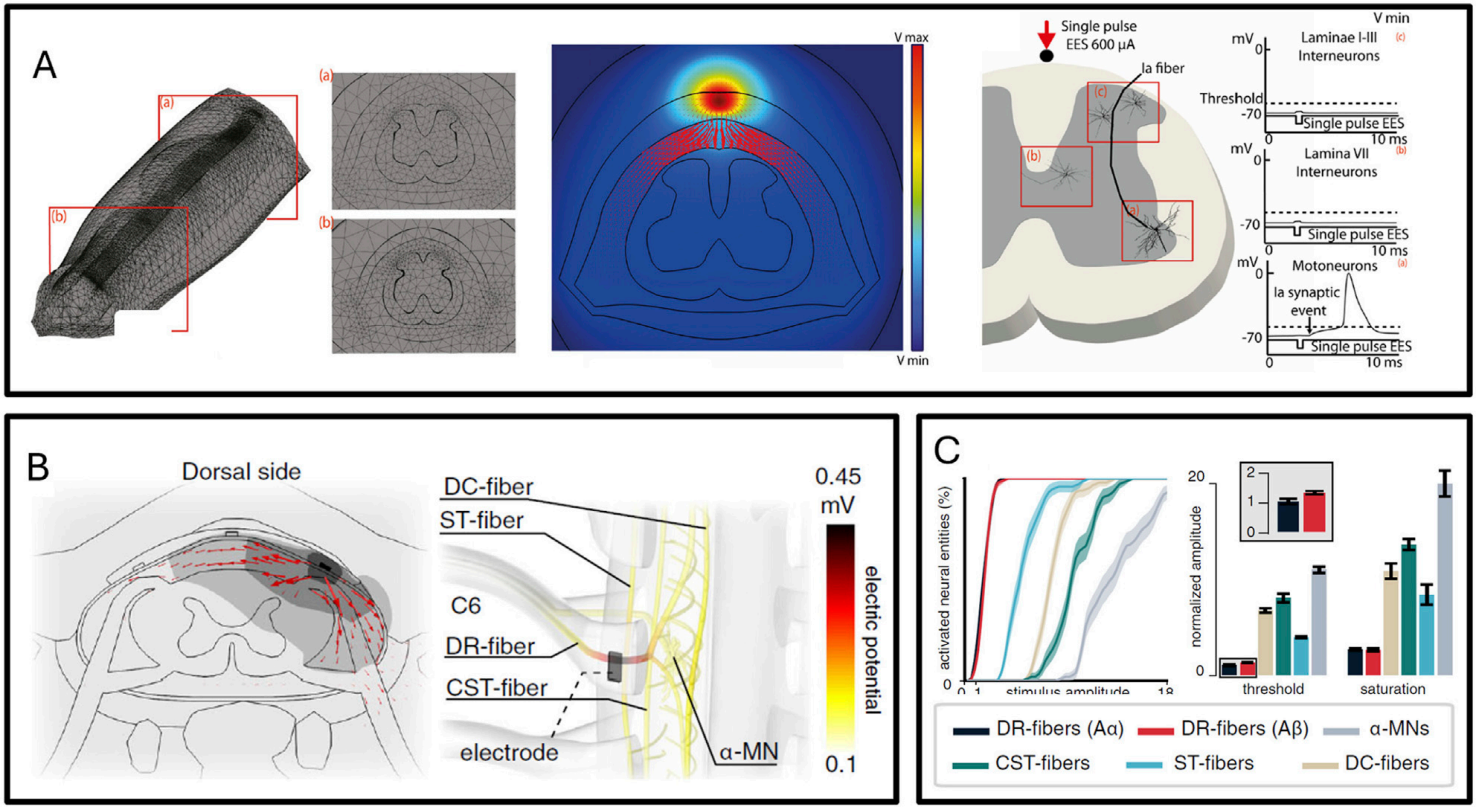
**(A)** Finite element simulation of lumbar spinal epidural electrical stimulation (Capogrosso et al., 2013). **(B)** Finite element simulation of cervical spinal epidural electrical stimulation (Greiner et al., 2021). **(C)** Different types of nerve fibers exhibit varying activation thresholds in the simulation (Greiner et al., 2021).

The team led by Grégoire Courtine extensively uses hybrid model simulation of dorsal root neuron activation under electrical stimulation to quantify epidural spinal stimulation (Capogrosso et al., 2013; Greiner et al., 2021). Figure 5A, B display the simulation model of spinal cord tissue interacting with electrodes, while Figure 5C illustrates that modeling and simulation can predict the targeted activation of different nerve fibers by electrical stimulation. Simulation technology can analyze the percentage of a type of neuron activated under certain intensity electrical stimulation at a certain location and the differences in activation of different types of neurons. Combined with the neural pathways above and below the neurons, the degree of activation of different spinal segments under certain positions and certain electrical stimulation intensities can be further deduced, and thus the degree of activation of different muscles can be inferred. The simulation model can help analyze the association of stimulation waveform, frequency, and intensity with walking behavior and upper limb motor behavior through the neural activation model and neural network dynamics model, thus rationally analyzing which form of stimulation can achieve the targeted recovery of motor functions.

### 3.3 Low invasive neuroelectrode prosthesis

The more stimulation sites, the higher the degree of invasiveness, and the stronger the targeting of activation, but this may also bring more serious biocompatibility and long-term stability issues (Alba et al., 2015; Du et al., 2017b; Günter et al., 2019). Researchers have conducted extensive evaluations of the performance of various electrodes through electrode implantation experiments in model animals and humans (Alba et al., 2015; Kozai et al., 2016). Current research indicates that semi-invasive electrodes, observed on the nerve’s exterior after implantation, show connective tissue hyperplasia, but human implantation experiments have proven that non-deforming cuff electrodes can maintain safety and stability for up to 7 years, and FINE, which causes nerve deformation, still maintains safety and stability after several years of use. For invasive electrodes, the electrode’s invasiveness directly damages nerve fibers, and the scar formation around the electrode affects the transmission of electrical stimulation. Additionally, inserting the electrode into the nerve may also cause inflammation and axonal demyelination. Besides, the implantation method for invasive electrodes is more complex. For flexible invasive electrodes, wire-guided implantation is necessary. Despite this, experiments have shown that mouse tissue maintains stability and compatibility with LIFE and TIME for several months (Std, 2007; Overstreet et al., 2019). This may be due to the scar formed after nerve damage preventing the transmission of nerve damage. The safe use time in the human body will be further extended.

By improving the structure and substrate materials of the electrodes, the biocompatibility of the electrodes can be enhanced (Raspopovic et al., 2021). First, the structural mechanical properties of the electrodes need to match the implantation location (Liu et al., 2019; Song K. I. et al., 2020). Cuff electrodes generally use flexible silicone resin or polyimide as the substrate, which can change shape as tissue grows after implantation (Zhang et al., 2019). Additionally, the entire electrode is commonly encapsulated with biocompatible polydimethylsiloxane film to enhance biocompatibility (Raspopovic et al., 2021).

**FIGURE 6:**
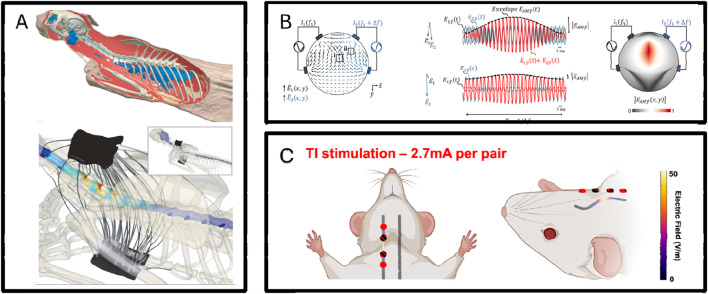
**(A)** Multi-electrode synergy enables non-invasive, non-destructive spinal stimulation in feline simulation models, with experimental validation confirming simulation reliability (Williams et al., 2022). **(B)** Temporal coherence stimulation demonstrate activation of deep brain neurons using extracranial electrodes, with experimental validation confirming simulation reliability (Grossman et al., 2017). **(C)** Temporal coherence simulations show that non-invasive electrodes can activate rat sublingual nerves, with experimental validation confirming simulation reliability (Missey et al., 2023).

Besides improving electrode manufacturing materials, low-invasiveness electrodes can also be enhanced in targeting through modeling as shown in Figure 6C (Wessel et al., 2023). In a study published in 2023, using temporal coherence stimulation and modeling simulation-assisted multi-electrode site cooperative action, effective stimulation of the sublingual nerve was achieved with low-intensity stimulation using electrodes fitted to the neck (Missey et al., 2023). Temporal coherence stimulation, besides being applied to peripheral nerve stimulation, has a wider application scenario in the cerebral cortex. A 2017 study as shown in Figure 6B showed that using temporal coherence stimulation can activate cortical cells under skin stimulation on the rat’s head (Grossman et al., 2017). Subsequent research has shown that temporal coherence stimulation can also play a role in larger animals such as humans (Esmaeilpour et al., 2021). Modeling methods can also accurately couple the cooperative action between different electrodes. In a 2021 study, modeling methods assisted in designing a stimulation method that can effectively activate neurons in the cat’s spinal cord from outside the body as shown in Figure 6A (Williams et al., 2022).

### 3.4 Peripheral nerve prostheses

Peripheral nerves are composed of bundles of nerve fibers and the epineurium that wraps these bundles. A nerve may contain multiple nerve bundles, each enveloped by a perineurium, with the epineurium wrapping multiple nerve bundles to form a peripheral nerve. Each nerve bundle contains multiple types of nerve fibers that can transmit ascending sensory or descending motor nerve signals. Different nerve bundles in a nerve may innervate and sense different organs, such as different muscles. To restore tactile sensation, electrical stimulation electrodes can be implanted on the outside or inside of a nerve. The degree of targeting achieved by the electrodes determines the precision of sensory restoration (Romeni et al., 2020).

Peripheral nerve electrodes come in various shapes, as shown in common implantation electrode diagrams (Sha et al., 2023; Sha and Du, 2024). Cuff electrodes (Christie et al., 2017) and flat interface nerve electrodes (FINE) (Freeberg et al., 2017) are non-invasive to nerves. Although FINE is also wrapped around the outside of the nerve, its cross-sectional shape is square, causing the nerve to be forced to deform. Through this method, the degree of separation between nerve bundles can be increased, enhancing the targeting activation of different bundles. Longitudinal intrafascicular electrodes (LIFE) (Overstreet et al., 2019), transverse intrafascicular multichannel electrodes (TIME) (Std, 2007), and the Utah Slanted Electrode Array (USEA) (Wark et al., 2013) are invasive. There are also various forms of non-invasive electrodes acting on the skin’s surface (Nonis et al., 2017; Missey et al., 2023).

Electrical stimulation simulation modeling can simulate the activation of nerve fibers by different electrode morphologies and can predict electrode morphology, electrode site arrangement, electrode implantation position, and electrical stimulation method in advance, thus providing a more rational way to design peripheral nerve electrodes. Depending on the implantation location, peripheral nerve interfaces are divided into nerve prostheses, autonomic nervous system nerve interfaces, and dorsal root ganglion interfaces, each with different application scenarios.

#### 3.4.1 Sensory-motor nerve prostheses

Peripheral sensory-motor nerve prostheses, also known as nerve prostheses, are advanced devices specially designed to restore the motor functions of amputees. Amputation directly affects the body’s motor ability, and wearing traditional prostheses cannot restore the perception of tactile sensation and the state of the prosthesis. This sensory loss affects the patient’s motor efficiency and accuracy. Nerve prostheses take into account the importance of somatosensory sensation, using feedback stimulation to reshape somatosensory sensation by stimulating residual peripheral nerves, thus providing more precise and natural motor control. Since the forms of amputation are diverse, nerve prostheses need to be personalized for different patients to adapt to their specific injury conditions. Specifically, the human motor system contains complex feedforward and feedback processing mechanisms: the motor center not only provides motor commands but also relies on various feedback information provided by the limbs, such as tactile and proprioceptive somatosensory sensations (Oddo et al., 2016). Somatosensory sensation plays a crucial role in the fine control of motor states, so effective nerve prostheses need to provide somatosensory information while reading motor commands.

Usually, amputees retain some muscles at the amputation site, and electromyography can read the state of these muscles, such as electromyography electrodes in an eight-shape, which can be used as motor commands to drive mechanical prostheses. At the same time, nerve prostheses contain sensors that detect their own state, such as surface pressure sensors and torque sensors at mechanical joints. The prosthesis control system can decode these signals into somatosensory neural stimulation signals and re-encode them into the nervous system through peripheral nervous system electrodes, achieving closed-loop control of nerve prosthesis input and output. Through continuous practice, patients wearing nerve prostheses can proficiently use mechanical arms and legs, with a smoothness far exceeding that of ordinary prostheses (Darie et al., 2017; Petrini et al., 2019).

Modeling and simulation technology can be used to optimize the selectivity of implanted peripheral nerve stimulation electrodes, predicting the nerve groups that can be activated by applying specific electrical stimulation at different implantation positions, thus optimizing the electrode structure, electrode implantation position, and electrical stimulation waveform. Besides, with the help of modeling and simulation, researchers can gain a deeper understanding of the principles of interaction between nerve prostheses and the nervous system, as most implantation effectiveness verifications are phenomenological and do not record whether the nerve fibers inside the nerves are effectively activated. Modeling and simulation can fill this gap, which has a driving effect on the understanding of many physiological characteristics (Raspopovic et al., 2012; Raspopovic et al., 2017; Zelechowski et al., 2020).

#### 3.4.2 Autonomic nervous system nerve interface

**FIGURE 7:**
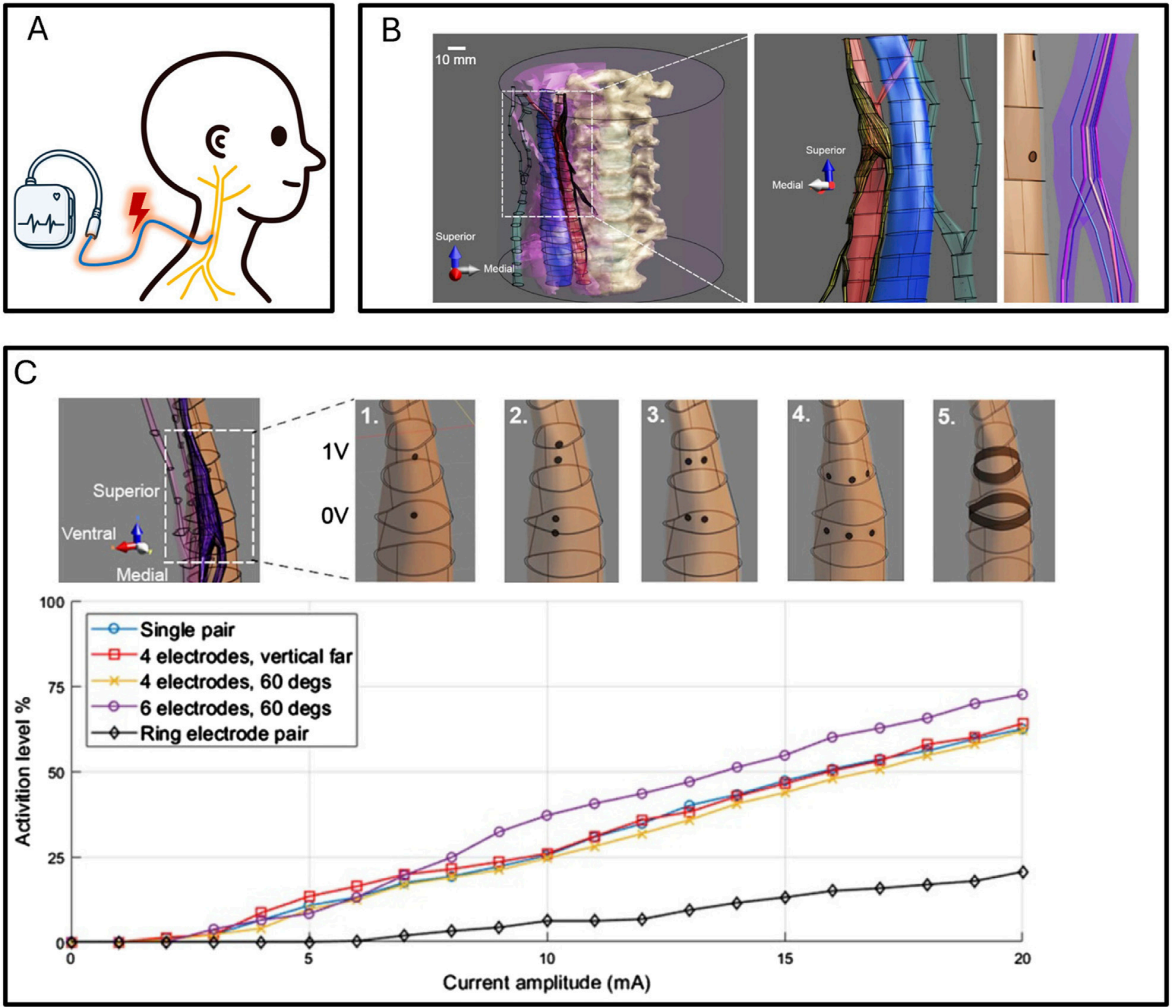
Vagus nerve stimulation Schematic and modelin **(A)** schematic diagram of vagus nerve stimulation. **(B)** Schematic diagram of finite element modeling of vagus nerve and surrounding tissues (Liu et al., 2023). **(C)** Effect of intravascular electrode geometry on vagus activation threshold (Liu et al., 2023).

In addition to the sensorimotor nerves, the peripheral nervous system contains a large number of autonomic nerves, such as the vagus nerve. The vagus nerve is a cranial nerve belonging to the parasympathetic nervous system, mediating various neurological functions such as heartbeat, breathing, blood pressure, inflammation, and even directly affecting brain function. Selective electrical stimulation of the vagus nerve has been widely used in anti-inflammatory treatments, epilepsy management, heart rate regulation, and blood pressure modulation (Wang Y. et al., 2021). Currently, finding a non-invasive, high-targeting method of vagus nerve stimulation is imperative. A novel approach uses an interventional method to implant stimulation electrodes inside the common carotid artery. Since the vagus nerve and the common carotid artery are both within the carotid sheath, their spatial positions are very close, so theoretically, intravascular electrical stimulation can affect the adjacent vagus nerve (Nicolai et al., 2023). To quantify this stimulation process, Liu used finite element modeling to analyze the stimulation behavior in animal models and human models before conducting human experiments to carefully assess the risks. In animal models, the actual stimulation intensity matched the results obtained from modeling simulation, indicating that simulations in human models can largely quantify the behavior of actual human body stimulation (Liu et al., 2023). Figure 7 provides a detailed display of the targeted differences caused by different implantation positions of the vagus nerve electrode. Besides, modeling simulation has also been applied to enhance the selectivity of vagus nerve stimulation, specifically using modeling simulation to guide the selective activation of certain branches in the vagus nerve bundle, thus achieving specific functional regulation without causing other side effects such as muscle reactions and heart rate deceleration (Plachta et al., 2013). Stephan L Blanz and others established a pig vagus nerve stimulation model, quantitatively analyzing the histochemical results of fiber types in the pig’s cervical vagus nerve and the relationship between electrically induced nerve activity recordings, deep cervical muscle activation, and heart rate changes. This study provides a reference framework for the design of vagus nerve electrodes, reducing the side effects of vagus nerve electrodes and improving the targeting of vagus nerve electrode stimulation (Blanz et al., 2023).

#### 3.4.3 Dorsal root ganglion nerve interface

The dorsal root ganglion is a group of nerve cells located in the intervertebral foramen of the spine, mainly containing the cell bodies of primary sensory neurons. These neurons are responsible for transmitting sensory information from the periphery to the central nervous system, including touch, pain, and temperature perception. Dorsal root ganglion stimulation has been proven to alleviate complex regional pain syndrome in some patients. Usually, the anode and cathode of the electrical stimulation electrode span the pedicle, and applying electrical stimulation at certain frequencies and intensities can activate the cell bodies or axons in the dorsal root ganglion, generating action potentials (Kolarcik et al., 2015). These action potentials enter the central system and interact with the neural circuits that mediate pain, thereby inhibiting chronic pain (Deer et al., 2019; Graham et al., 2022b; Abd-Elsayed et al., 2024; Graham et al., 2022a).

**FIGURE 8:**
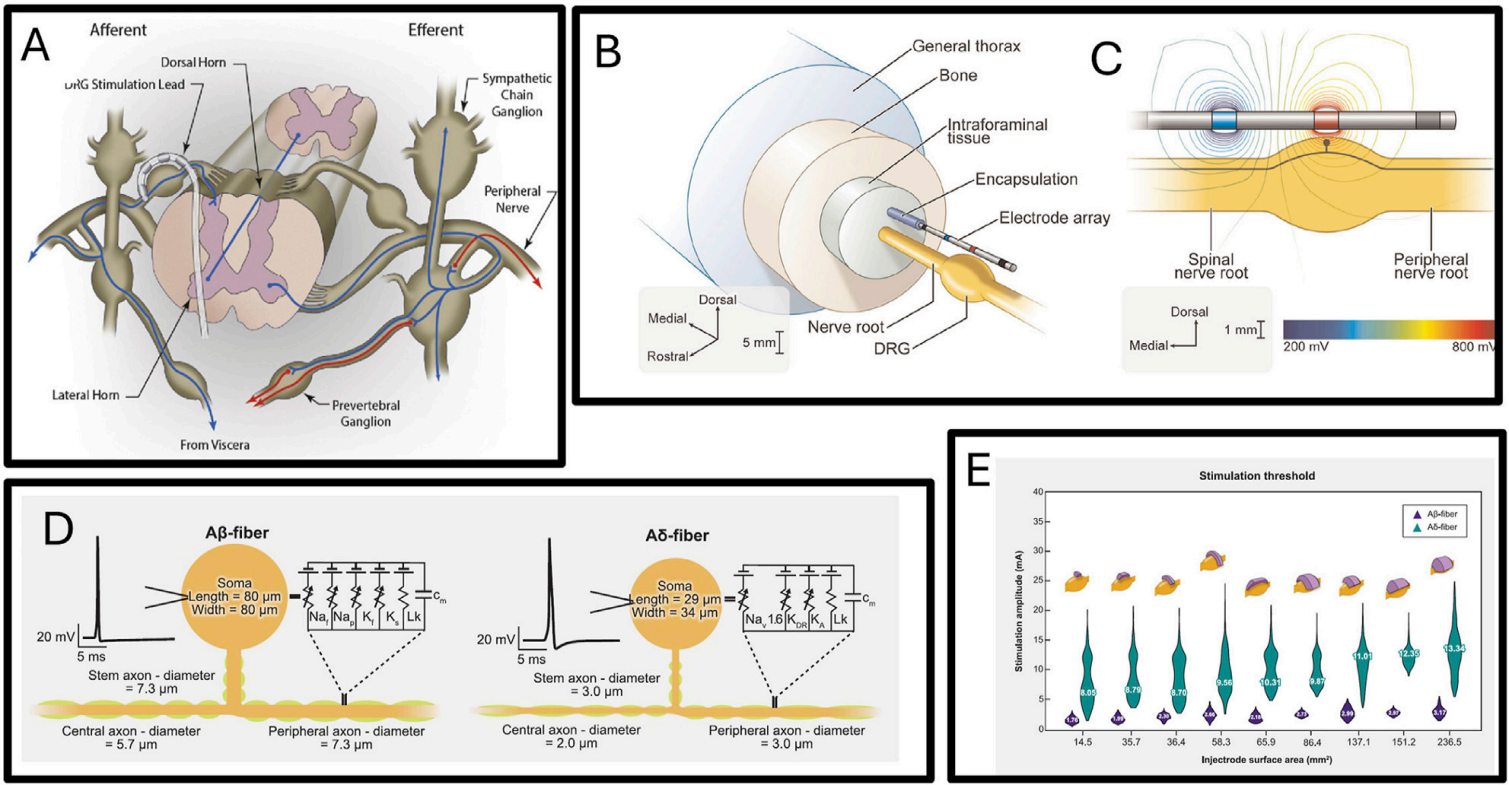
**(A)** Diagram of dorsal root ganglion stimulation electrode implanted in foramen interbod (Sverrisdottir et al., 2020). **(B)** Schematic diagram of three-dimensional finite element model of dorsal root nerve stimulation (Graham et al., 2022a). **(C)** Isopotential lines of the extracellular voltages generated by bipolar DRGS calculated from the FEM (Graham et al., 2022a). **(D)** Multi-compartment models of two types primary sensory neurons (Bhowmick et al., 2024). **(E)** The distribution of activation thresholds of A*β*- and A*δ*-fibers generated by the various Injectrode geometries with the mean values inset and the corresponding Injectrode geometry at the top of each violin plot (Bhowmick et al., 2024).

Since the dorsal root ganglion contains a wide variety of nerve types, such as myelinated Aα, Aβ, and Aδ fibers, and unmyelinated C fiber neurons (several subgroups), it is impossible to directly determine which type of nerve is activated by dorsal root ganglion stimulation, Figure 8A shows the implantation of a human dorsal root stimulation electrode. so the mechanism of dorsal root ganglion electrical stimulation is not yet clear. Simulation models provide an excellent platform for researching the analgesic mechanism of dorsal root ganglion electrical stimulation. Dorsal root ganglion electrical stimulation simulation models can help researchers determine which type of nerve is activated under specific forms of stimulation, Figures 8B–D show the modeling of the dorsal root tissue environment and the modeling of the nerves contained within, thus determining the mechanism by which dorsal root ganglion stimulation functions. Scott F. Lempka and others published a study in 2019 showing that computational models indicate that dorsal root ganglion stimulation in clinical stimulation scenarios drives the activity of Aβ neurons, not affecting C neurons (Graham et al., 2019). This suggests that dorsal root ganglion stimulation may alleviate pain by repeatedly activating large myelinated afferent nerves, activating the pain gate mechanism in the dorsal horn, thereby relieving pain. C-type primary sensory neurons mediated by higher frequency signals may be closely related to chronic pain. David B. Jaffe’s computational study in 2015 showed that the unmyelinated C-type neuron’s pseudo-bipolar structure at the axon-soma junction has low-pass filtering properties, suggesting that C-type neurons at the dorsal root ganglion may have a role in filtering pain signals (Sundt et al., 2015). A 2018 study on dorsal root ganglion electrical stimulation simulation proved that electrical stimulation can enhance the filtering properties of C-type primary sensory neurons in the DRG, which may be one reason for the analgesic effect of dorsal root ganglion stimulation (Kent et al., 2018). Besides researching the mechanism of action of dorsal root ganglion stimulation, modeling and simulation have also been applied to explore reasonable stimulation parameters. In 2024, Scott F Lempka and others used a computational model to assess the feasibility of the injectrode system designed in their 2021 study for human scenarios (Bhowmick et al., 2024). This study’s model was based on the experimental results of previous injectrode experiment (Dalrymple et al., 2021) and developed multiple human-scale computational models of DRG stimulation to study how design parameters such as injectrode size and orientation affect nerve activation thresholds. The simulation matched acute animal experiment measurements, and the human model showed that by adjusting the injection and stimulation parameters, the Injectrode system can activate large diameter afferent nerves (Aβ fibers) without activating pain-related mechanoreceptors (Aδ fibers). The simulation results are displayed in Figure 8E.

### 3.5 Retinal nerve interface

**FIGURE 9:**
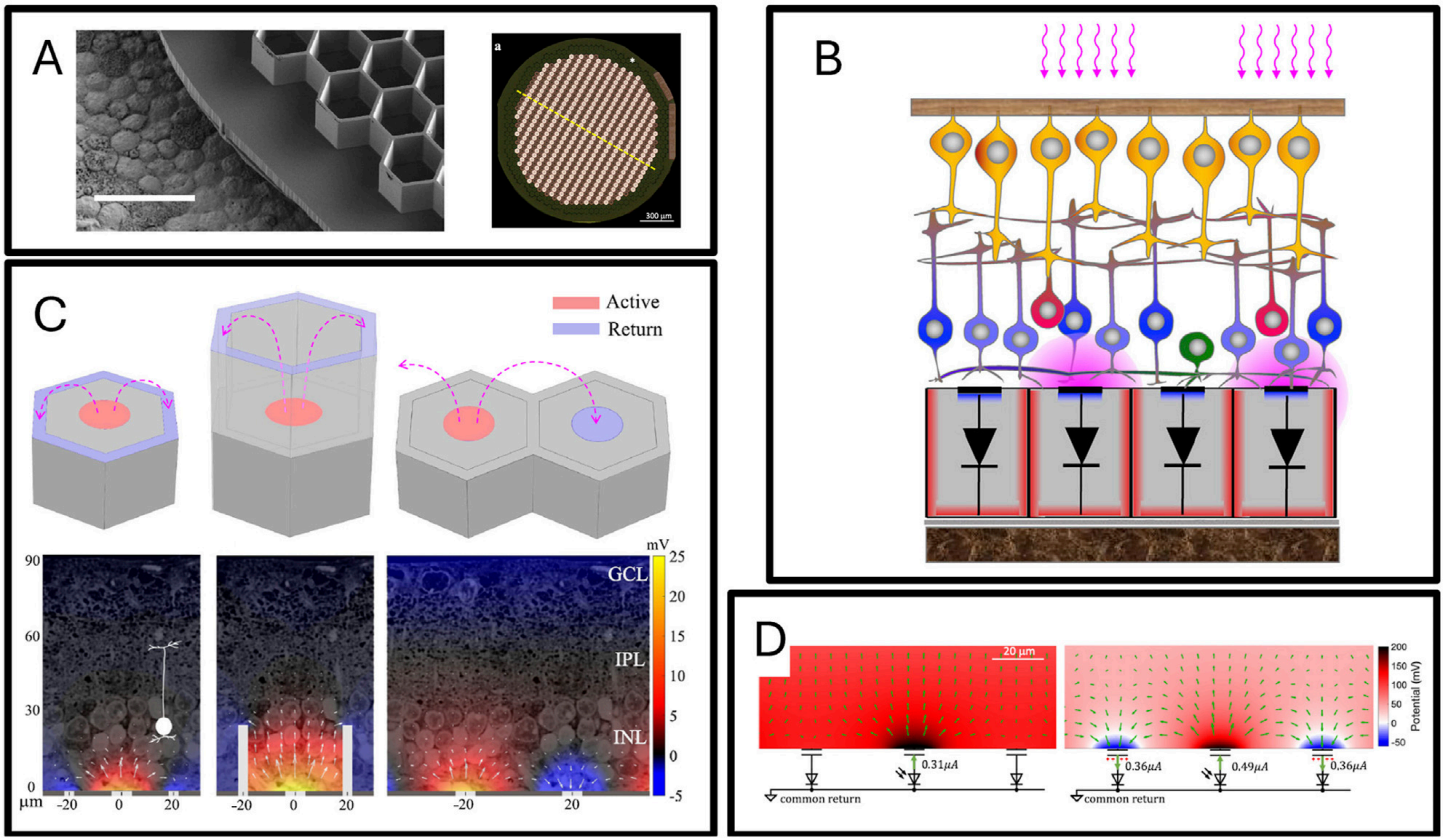
**(A)** Honeycomb retinal neuroprosthesis imagery (Liu et al., 2023). **(B)** Operational principles of retinal neuroprostheses (Liu et al., 2023). **(C)** Simulation of electric field variations induced by electrodes in honeycomb retinal neuroprostheses (Liu et al., 2023). **(D)** Phototransduction and multi-site collaboration mechanisms of retinal neuroprostheses (Wang et al., 2022).

Visual nerve prostheses mainly include retinal nerve prostheses and cortical visual prostheses. Research on retinal nerve prostheses has been relatively mature, having been applied for nearly half a century (Greenberg et al., 1999), and is used in conditions such as pigmentary retinopathy and age-related macular degeneration-induced visual defects (Loizos et al., 2018). For patients with age-related macular degeneration, since cone and rod cells may degenerate or be lost due to disease, a viable strategy to restore their visual function is to implant a retinal nerve prosthesis under the retina to activate bipolar cells. The implantation site is usually below the retina at the central fovea, and a small space needs to be created inside the eyeball during the surgical process to accommodate the implant. In retinal nerve prostheses, the image captured by the camera uses pulsed near-infrared light projected from augmented reality glasses onto the subretinal photovoltaic array, where each pixel’s photodiode converts incident photons into pulsed currents flowing through the tissue between the array’s active stimulation electrodes and return electrodes, polarizing bipolar cells (Li and Li, 2015). The degree of restored vision is not only limited by the spatial resolution of stimulation (pixel size) but also by the contrast (degree of electric field dispersion). Generally, a site is composed of a central stimulation site and surrounding return sites. For sites distributed on a plane, continuously increasing the arrangement density will increase the stimulation’s spatial resolution but will reduce the stimulation’s contrast, as the vertical dispersion of the electric stimulation field is limited, reducing the distance between the central stimulation site and surrounding return sites will not only activate the directly opposing bipolar cells but also the adjacent bipolar cells. Daniel Palanker and others proposed a 3D honeycomb structure electrode array in 2019 to address this issue, where the return sites of this electrode are higher than the stimulation sites (Flores et al., 2019) as shown in Figure 9A. The study based on finite element models transformed the simulated electric fields produced by the device into responses of neurons inside the retina, and detailed comparisons were made between the electric field differences and the activation differences of bipolar cells during electrical stimulation between this 3D honeycomb structure electrode and flat electrodes. Through modeling and simulation, it was proven that this 3D honeycomb-shaped electrode can significantly reduce the threshold for stimulating bipolar cell activation, decoupling the stimulation penetration depth and pixel width, and the current density at the stimulation threshold of the honeycomb electrode does not increase significantly with the reduction in pixel size. Since it cannot be completely guaranteed that bipolar cells can migrate into the grooves of the 3D honeycomb-shaped electrode, in a subsequent study published in 2022, Daniel Palanker and others modulated the electrical stimulation method, achieving vertical control of the activation electric field on the planar photovoltaic electrode array as shown in Figures 9B–D (Wang et al., 2022). The specific method involved temporal modulation of electrical stimulation between pixel points, using surrounding stimulation sites as return sites through circuit and lighting protocol control. Researchers constructed an anatomically accurate rat eye model to simulate the potential produced by the subretinal implant on the cornea, and the results proved that this local charge balance limited the lateral diffusion of the electric field, reducing crosstalk. Animal experiments further verified the accuracy of the model prediction, proving that vertical control of the activation electric field can also be achieved in planar retinal photovoltaic prostheses.

### 3.6 Cochlear implant

The cochlear implant is a complex implanted hearing aid designed to help people with profound deafness or severe hearing impairment restore hearing. The device simulates the function of the cochlea, where an external sound reception facility receives sound signals, and an electronic control system re-encodes the sound signals into electrical stimulation signals, which are then used to stimulate the auditory nerve to restore hearing. Cochlear implants are the most implanted neural prostheses and are widely used in the field of restoring hearing impairments caused by middle ear damage (Lei et al., 2021). Although cochlear implants have been proposed and applied for over half a century, despite the very refined design of modern cochlear implant electrode arrays, cross-activation between electrodes (channel interaction) may still occur. This phenomenon can affect the resolution and clarity of sound, especially in complex auditory environments. Additionally, the degree of contact between the electrodes and neural tissue is also a major factor affecting the effectiveness of cochlear implants. If there is insufficient contact between the electrodes and nerve fibers, it may lead to low electrical stimulation efficiency, requiring higher energy to achieve the same auditory induction, which may accelerate battery consumption or produce greater stimulation to surrounding tissues. Electrode insertion may cause inner ear damage, including the remaining hearing cells, which could lead to further hearing loss for patients who still have partial hearing.

**FIGURE 10:**
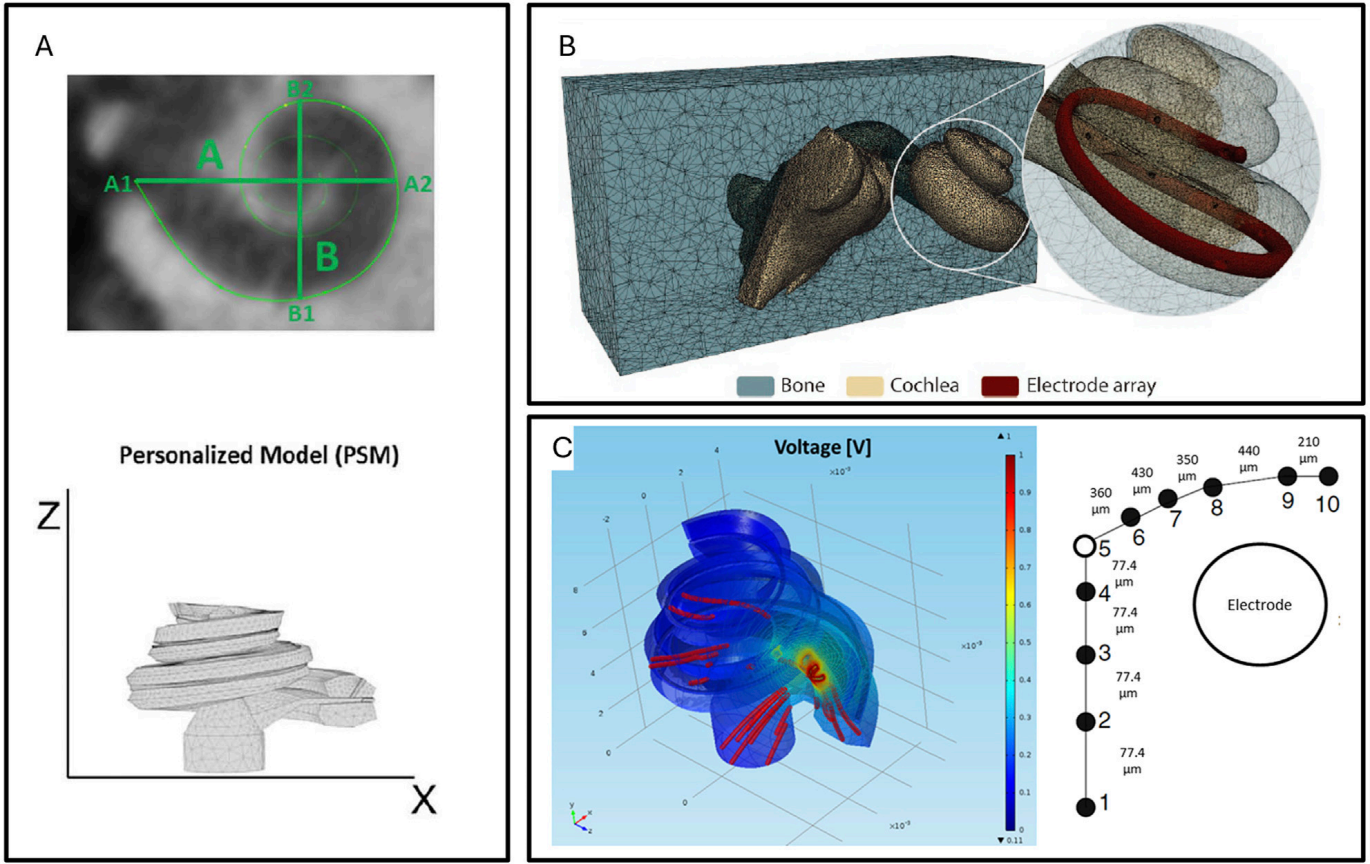
**(A)** Reconstruction of the cochlea’s 3D model using actual CT data (Nogueira et al., 2016). **(B)** Creation of a realistic model of a hearing aid electrode implanted in the cochlea using 3D software (Gerber et al., 2017). **(C)** Finite element electromagnetic simulation to model the changes in the electrical field within the cochlea induced by electrical stimulation (Nogueira et al., 2016).

An application of modeling and simulation in cochlear implants is the ability to predict the speech produced by cochlear implants. Simulation models predict the distribution of the electric field produced by electrical stimulation in the cochlea, thus guiding the design of the electrodes, the relative implantation position of the electrodes and the cochlea, and rationally conjecturing how to coordinate the stimulation voltage and waveform at multiple sites to produce more accurate sounds. Finite element modeling of cochlear implants was proposed in the 1990s (Frijns et al., 1995; Agrawal and Newbold, 2012; Pearl et al., 2014), but these studies provided detailed theoretical analyses of the interaction between cochlear implants and the cochlea at the theoretical level. Until around 2015, parameterized cochlear implant models began to be applied (Malherbe et al., 2016; Wong et al., 2016). Parameterized models, through individual CT imaging, realistically establish the structure of the implanted electrodes in the cochlea in the model, greatly enhancing the model’s accuracy and truly being predictive models. Figure 10 illustrates the process from the cochlear CT scan in Figure 10A to establishing the simulation model in Figure 10B, and obtaining the simulation results in Figure 10C. By coupling finite element models, auditory nerve models, and sound coding models step by step, the relationship between the electrode’s electrical stimulation method and the individual’s hearing produced by the implanted cochlear implant can be established, which is crucial for improving cochlear implant parameters and enhancing its speech performance (Nogueira et al., 2016; Gerber et al., 2017; Castle et al., 2023).

Cochlear finite element model simulation can also guide the optimal implantation position of cochlear implants. In the implantation of cochlear implants, both the depth of insertion and the proximity of the electrode to the cochlear wall affect safety and cochlear implant performance. In a 2022 study, Enver Salkim and others analyzed the impedance changes at different positions during the electrode implantation process through a parameterized cochlear implant model (Salkim et al., 2022), establishing the relationship between impedance changes and the process of the electrode approaching the cochlear wall during insertion. This may have clinical value for assessing electrode positioning.

## 4 Discussion

### 4.1 Conclusion

Today, neurophysiological modeling and simulation technology have provided significant support for the design and implantation of various neural prostheses, accelerating the development of neural prostheses and providing theoretical guidance for effective interactions between various neural prostheses and the nervous system. Especially using modeling technology, more refined stimulation control can be assisted, gradually replacing high-invasive electrodes with lower-invasive electrodes, multi-channel, and temporal coherence stimulation methods.

### 4.2 Enhancing model prediction accuracy

However, simulation models cannot perfectly predict the interaction between electrodes and tissues. The main reason is the model’s simplification of the dielectric properties of physiological tissues and the simplification of neuron modeling, resulting in differences between model simulation predictions and actual experimental results.

Specifically, the simplification of the dielectric properties of physiological tissues mainly comes from the following points: first, the neglect and simplification of tissue boundaries, treating them as a single tissue type and ignoring the differences in their conductivity and permittivity. Second, the simplification of the existence of small tissues, such as many small tissues like blood vessels and lymphatic vessels, which are ignored in the modeling process. Third, the simplification of the tensor parameters of tissue conductivity and permittivity. In reality, the conductivity and permittivity of each position in each tissue are anisotropic, especially for tissues like white matter. Due to the presence of nerve fiber bundles, the conductivity and permittivity of the tissue vary greatly depending on whether it is parallel or perpendicular to the direction of the fiber bundles. Theoretically, an accurate description requires defining the conductivity and permittivity tensor of the tissue occupying space at each position, but to reduce the modeling difficulty, the anisotropic conductive and dielectric properties of the tissue are generally ignored. For example, when establishing a model of brain white matter, the anisotropic conductivity of the brain white matter occupying space is not set according to the direction of the fiber bundles, but the tissue is treated as an isotropic conductor. The simplification of the model has the following reasons: one is to reduce the modeling complexity and the difficulty of model simulation; the other is because there is no non-invasive high-resolution method to obtain the information needed to establish a complex model. With the development of various non-invasive high-resolution imaging techniques such as micro-CT, micro-MRI, and micro-US, these imaging techniques can provide richer information, which will undoubtedly promote the progress of modeling and simulation. Additionally, with the development of the field of computer vision, many tissue three-dimensional reconstruction programs are gradually becoming simpler, which will also simplify the tedious procedures in the modeling process. At the same time, the annual improvement in computer computing power also supports the simulation of larger-scale, higher-complexity models, so there is reason to believe that the accuracy of modeling and simulation will rapidly increase.

The finite element model of neurons is more accurate than the MRG model. Computational complexity and modeling process difficulty limit the application of the neuron’s finite element model. In addition, the inertia of using the neuron multi-compartment model for a long time also indirectly limits the popularity of the neuron’s finite element model. At present, computer computing power has reached a very high level, and some relatively complex neuron finite element models can also be solved relatively quickly, but because there are too many program environments and literature environments supporting the neuron MRG model, and much less support for the neuron finite element model, most researchers will not choose to use the neuron’s finite element model for neurophysiological simulation. Besides, establishing a neuron’s finite element model requires constructing a watertight three-dimensional structure of the neuron, which is relatively simple for peripheral nervous system neurons, which basically have no branching, but much more difficult for complex-shaped central neurons. This is because there is a lack of sufficient data to support the reconstruction of the complex irregular surfaces of neurons, and reconstructing the entire neuron’s complex irregular surface itself requires support from high-performance image processing equipment. At present, with the development of cross-scale imaging technology, many studies provide more abundant raw data needed for neuronal shape reconstruction. In the future, more open-source datasets of neuronal structures will become available, offering increasingly precise and detailed information on neuron morphology. Additionally, new algorithms and software solutions will emerge to facilitate the conversion of neuronal skeletons into watertight models, making finite element modeling of neurons simpler and more accessible. With improvements in computational power, along with advancements in neuronal datasets and reconstruction algorithms, the use of finite element models—offering more accurate neuronal representations—will become increasingly widespread.

### 4.3 Enhancing the usability of models

In the future, the simulation and modeling of neural electrodes are expected to become standard practices in clinical electrode implantation and design. However, the widespread adoption of these processes is currently hindered by the high technical barriers of simulation modeling, limiting its use. This is because effective simulation modeling requires not only a solid background in biology but also proficiency in mathematics, physics, computer science, mastery of one or more programming languages, and the ability to use various automated or semi-automated tools for image segmentation and 3D modeling. Most clinicians and neuroscientists, due to their lack of expertise in these areas, struggle to independently build simulation models to predict or guide electrode development and clinical applications. There is an urgent need for the development of an open-source, visually guided, non-programming-based modeling method. Current open-source modeling processes are mostly programming-based, and only a few models offer graphical interfaces. Many of these tools are not fully open-source, and some modules are costly. In the future, modeling and simulation tools must reduce technical barriers and become more user-friendly, especially for non-programmer users such as clinicians and neuroscientists who require neural prosthetics. The following suggestions can help achieve this goal. (1) Develop a graphical user interface: Create an intuitive interface that allows users to build and simulate models through visual methods, without the need for complex programming knowledge. (2) Integrate artificial intelligence: Use AI algorithms to automatically optimize simulation parameters, reducing manual input. AI could also offer interactive features, allowing users to guide the model framework through language, refining simulation details. (3) Provide educational resources: Develop clear online tutorials and documentation to help users learn how to utilize these tools. (4) Modular design: Enable users to customize model components based on their needs, streamlining the modeling process. (5) Promote open-source protocols: Adopt an open-source software model, encouraging global developers to contribute to the improvement and updating of these tools. Through these methods, neural electrode modeling and simulation tools will become more user-friendly, facilitating the broader adoption of clinical electrode implantation and advancing research in electrode development.
